# Analysis of the clinical value of triiodothyronine levels in sudden sensorineural hearing loss: A retrospective observational study

**DOI:** 10.1097/MD.0000000000043843

**Published:** 2025-08-15

**Authors:** Dachun Dai, Yuehua Qiao, Baihe Zhang, Yating Wang, Mengyuan Zhao, Jie Huang

**Affiliations:** a Department of Otorhinolaryngology, Taikang Xianlin Drum Tower Hospital, Affiliated Hospital of Medical School, Nanjing University, Nanjing, Jiangsu, China; b The Artificial Hearing Laboratory of Jiangsu Province, The Institute of Audiology and Speech Science of Xuzhou Medical College, Xuzhou, Jiangsu, China; c Public Administration, Nanjing Medical University, Nanjing, Jiangsu, China; d Department of Otorhinolaryngology and Head and Neck Surgery, BenQ Medical Center, The Affiliated BenQ Hospital of Nanjing Medical University, Nanjing, Jiangsu, China; e Fourth Clinical Medical College, Nanjing Medical University, Nanjing, Jiangsu, China.

**Keywords:** low triiodothyronine syndrome, normal thyroid syndrome, sudden sensorineural hearing loss, thyroid dysfunction, thyroid hormone

## Abstract

Thyroid hormone (TH) plays a key role in neurological and metabolic regulation. However, its association with sudden sensorineural hearing loss remains unclear. This study aimed to investigate the potential role of triiodothyronine (T_3_) in sudden sensorineural hearing loss (SSNHL) and its relationship with emotional and sleep disturbances. We retrospectively analyzed 88 patients with SSNHL and 32 patients with chronic otitis media as controls. SSNHL patients were stratified into 3 audiometric subgroups. Data collected included demographics, TH status profiles (T_3_, free triiodothyronine [FT_3_], thyroxine, thyroid-stimulating hormone), and assessments of anxiety, depression, and sleep quality. Abnormal TH levels were observed in 34.1% of SSNHL patients, with decreased T_3_ and FT_3_ levels in 27.3% and 18.2%, respectively – significantly higher than in the chronic otitis media group (6.3%). FT_3_ levels differed significantly between the low- and total-frequency subgroups. The prevalence of anxiety, depression, and sleep disorders among SSNHL patients was 42%, 33.3%, and 51.1%, respectively, significantly exceeding that in controls. Most patients with low T_3_ syndrome experienced spontaneous normalization of hormone levels, unlike those with hypothyroidism. SSNHL may be associated with transient TH alterations, particularly reductions in active T_3_. Monitoring thyroid function and addressing psychosomatic factors may be important in managing SSNHL.

## 1. Introduction

Triiodothyronine (T_3_) is the most biologically active Thyroid hormone (TH) and plays a critical role in regulating cellular energy metabolism and neurovascular function. Among all organs, the brain – due to its high metabolic demands – maintains the highest tissue ratio of T_3_/thyroxine (T_4_; 0.5).^[1]^ Positron emission tomography has demonstrated that hypothyroidism is associated with significantly reduced regional cerebral blood flow and glucose metabolism.^[2]^ Recent studies have shown that abnormalities in TH levels may also contribute to vestibular symptoms such as vertigo and have been implicated in the pathogenesis of sudden sensorineural hearing loss (SSNHL).^[3,4]^ However, the specific role of T_3_ in SSNHL remains poorly understood. In particular, it is unclear whether decreased T_3_ levels are associated with auditory dysfunction alone, or whether they also relate to common comorbid symptoms such as anxiety, depression, and sleep disturbances. Therefore, the study aims to investigate T_3_ abnormalities in patients with SSNHL and explore their potential links to both hearing outcomes and emotional/sleep status.

## 2. Materials and methods

### 2.1. Patients

Patients were admitted after being diagnosed with SSNHL according to clinical practice guidelines.^[5]^ Patients with chronic otitis media (COM) were chosen based on the feasibility of clinical recruitment and their comparable audiological evaluation environment within the same hospital system.

Inclusion criteria: Patients with the initial onset of unilateral SSNHL within 1 to 14 days; Hearing loss (HL) of 20 dB over 2 consecutive frequencies in average air conducted pure tone auditory threshold (0.5, 1, 2, 4 kHz). Patients who agreed to participate and provide informed consent. Exclusion criteria: Patients with serious systemic diseases, diabetes, and hypertension in unmanageable states, epilepsy, psychosis, ulcers of the active digestive tract, hemorrhagic disease, or any other contraindications to hormone therapy.

This study was approved by the Medical Ethics Committee of the Benq Hospital Affiliated to Nanjing Medical University, and written informed consent was obtained from all participants prior to data collection.

### 2.2. Methods

General demographic and clinical data were collected, including sex, age, HL level, and presence of vertigo. The following variables were assessed both before and after treatment: pure tone averages at 0.5, 1, 2, 4 kHz; thyroid function (T_3_, T_4_, free triiodothyronine [FT_3_], free thyroxine [FT_4_], thyroid-stimulating hormone [TSH], thyroid peroxidase antibody, thyroglobulin antibody [TG-ab], and thyrotropin receptor antibody levels); and psychological measures of anxiety, depression, and sleep quality. To investigate frequency-specific associations, SSNHL patients were divided into 3 groups based on their audiogram patterns: low frequency group (subgroup L), total-frequency group (subgroup T), and high frequency group (subgroup H). This stratification was pre specified according to established audiological classification criteria outlined in the 2015 Chinese Guidelines for Sudden Deafness^[5]^ and was determined prior to statistical analysis. Subgroup L was defined by HL primarily at low frequencies (0.5–1 kHz), and subgroup H was defined by HL primarily at high frequencies (2–4 kHz).

### 2.3. Treatment protocol

All SSNHL patients received of treatment in 14 days. Intravenous drugs were administered in the first 5 days: methylprednisolone (40–80 mg, 1 mg/kg/d) and a combination of gingko biloba extract 87.5 mg qod and mecobalamine 0.5 mg qod. HL more than 70 dB was treated combined with batroxobin (5 U qod, 6 times), sequential intratympanic drug therapy (dexamethasone 0.15 mg qod, 6 times) and hyperbaric oxygen therapy.^[5–7]^

### 2.4. Classification of thyroid dysfunction

Patients were diagnosed with low T_3_ syndrome when there was a decrease in T_3_ and/or FT_3_ levels with or without a decrease in T_4_ levels and without an increase or a decrease in TSH levels. Patients were diagnosed with hypothyroidism when a decrease in T_4_ and/or FT_4_ levels and an increase in TSH levels were observed, but they were diagnosed with subclinical hypothyroidism only if their TSH level increased.

### 2.5. The criteria for assessing anxiety and depression

The degrees of severity of anxiety and depression in the patients were assessed using the Hamilton rating scale for depression (HAMD).^[8]^ Patients were considered anxious if the total HAMD-17 score was greater than or equal to 7. A total HAMD score greater than or equal to 7 indicated depression.

### 2.6. The sleep assessment criteria

The Pittsburgh Sleep Quality Index (PSQI) was used to assess sleep: 10 ≥ PSQI ≥ 0 indicated good sleep, and 21 ≥ PSQI ≥ 11 indicated a sleep disorder.

### 2.7. Statistical analysis

All statistical analyzes were performed using SPSS 26.0 software (IBM Corp., Armonk). Continuous variables with a normal distribution are reported as means ± deviations (SD), and categorical variables are presented as frequencies (%). Group comparisons were conducted using the chi-square test (χ^2^), independent-sample *t* test, Fisher’s exact test, 1-way ANOVA followed by LSD post hoc test, as appropriate.

To evaluate the associations between demographic and TH variables and the type of SSNHL (low-, total-, or high frequency), multinomial logistic regression was employed. This model was selected because the outcome variable is nominal with 3 unordered categories, making it more suitable than binary logistic regression or ordinal models for capturing subtype-specific associations. Age, sex, and low T_3_ levels were included as covariates in the model.

Multicollinearity among predictors was assessed using variance inflation factor values. All variables had variance inflation factor values <2.0, indicating no significant multicollinearity. Only cases with complete data across all variables of interest were included; thus, complete case analysis was performed, and no imputation methods were applied. A *P*-value of <.05 was considered to indicate a statistically significant difference.

## 3. Results

### 3.1. General situation and hearing outcomes of patients

A total of 88 SSNHL patients and 32 COM patients were enrolled. Forty-two cases occurred in the left ear and 46 cases occurred in the right ear in the SSNHL group (Table 1).

**Table 1:** Correlation analysis of the general situation and hearing outcome of SSNHL.

Group	Sex		Age (mean ± SD, yr)	Affected ear		Vertigo	PTA (mean ± SD dB HL)			*P*
	M	F		L	R		Pre-therapy	Posttreatment	Hearing improvement	
COM (n = 32)	13	19	41.23 ± 17.30	17	15	0				.05
SSNHL (n = 88)	34	54	46.91 ± 14.25	42	46	16				
Subgroup L (n = 31)	5	26	38.16 ± 10.50	14	17	6	60.44 ± 11.55	30.26 ± 18.43	31.26 ± 21.34	
Subgroup T (n = 42)	22	20	54.45 ± 15.40	22	20	10	88.78 ± 22.45	70.52 ± 25.43	20.23 ± 19.25	
Subgroup H (n = 15)	7	8	47.60 ± 12.95	6	9	0	86.77 ± 21.08	71.49 ± 26.74	17.02 ± 8.93	
*P* (P_s_: *P* subgroup)	*P* < .05		P_s_ < .05 LSD *P*_T-H_ > .05	*P* > .05		*P* > .05	P_s_ < .05 LSD *P*_T-H_ > .05		P_s_ < .05 LSD *P*_T-H_ > .05	

COM = chronic otitis media, F = female, L = left ear, M = male, PTA = pure tone audiometry, R = right ear, SD = standard deviation, SSNHL = sudden sensorineural hearing loss.

### 3.2. Age distribution

No significant differences were observed between the SSNHL group and the COM group. There was a difference between 3 subgroups, but no difference in subgroup T and subgroup H after the LSD test. The mean age of subgroup T and subgroup H were 54.45 ± 15.40 years and 47.60 ± 12.95 years.The minimum mean age was in subgroup L, with an average age of 38.16 ± 10.50 years.

### 3.3. Sex distribution

There were 54 males and 34 females in SSNHL, there were 19 men and 13 women in COM group. No significant differences between the 2 groups.A female sex pre-dilection for the disease was observed in subgroup L. Only 5 patients were men and the male to female ratio was 0.19. The male to female ratio in these groups was approximately 1.0 in subgroup L and subgroup H. Significant differences were observed between subgroup L and the other 2 subgroups.

### 3.4. Vertigo as a complication

Six patients with SSNHL in subgroup L had vertigo and 10 patients in subgroup T had vertigo. No patients in subgroup H and COM had vertigo.

### 3.5. Hearing improvement after treatment

In this study, an average hearing improvement of 31.26 ± 21.34 dB HL was achieved in subgroup L, and improvements of 20.23 ± 19.25 dB HL and 17.02 ± 8.93 dB HL were achieved in subgroup T and subgroup H, respectively. Differences between groups were significant, but differences between subgroup T and subgroup H were not significant after the LSD analysis.

### 3.6. Status of thyroid hormone and mood and sleep states

#### 3.6.1. Status of mood and sleep

Approximately 42.0% of the patients with SSNHL had anxiety, 33.0% had depression, and 51.1% had sleep disorders. The incidence rates of anxiety, depression, and sleep disorders in COM were 21.9%, 12.5%, and 15.6%. In subgroup L they were 45.2%, 25.8% and 64.5% respectively. The incidence rates in subgroup T were 42.9%, 38.1%, and 50.0%, respectively, and those in subgroup H were 33.3%, 33.4%, and 26.7%, respectively. Significant differences were observed between SSNHL and COM, not among the 3 SSNHL subgroups.

#### 3.6.2. Status of thyroid hormone profiles

TH abnormalities were significantly more common in SSNHL patients (34.1%) than in COM controls (6.3%; *P* < .05). The prevalence of abnormal TH values in the SSNHL subgroups was 32.3% in subgroup L, 35.7% in subgroup T, and 33.3% in subgroup H, with no significant differences among the 3 subgroups (Table 2).

**Table 2:** Comparison of thyroid hormone in SSNHL patients with different hearing loss.

			Subgroup L (31)	Subgroup T (42)	Subgroup H (15)	Total	*P*
TH abnormity			15 (32.3%)	15(35.7%)	5 (33.3%)	30 (34.1%)	χ^2^ = 0.099 *P* > .05
T_3_↓	Pre-therapy	n	8	14	2	24 (27.3%)	χ^2^ = 2.477 *P* = .290
		Mean ± SD	1.11 ± 0.13	1.01 ± 0.16	1.20 ± 0.85	1.08 ± 0.14	P_L/T_ (independent-sample *T*-test *t* = 2.852 *P* > .05)
	Posttreatment n		1	6	0	7	Fisher *P* = .192
FT_3_↓	Pre-therapy	n	4	11	1	16 (18.2%)	χ^2^ = 3.956 *P* > .05
		Mean ± SD	1.84 ± 0.18	1.68 ± 0.25	1.87	1.73 ± 0.23	P_L/T_ (independent-sample *T*-test *t* = 3.179 *P* = .002)
	Posttreatment n		1	4	0	5	Fisher *P* = .475
T_4_↓	n		0	5	1	6	Fisher *P* = .139
	Mean ± SD			3.90 ± 1.64	4.35	3.47 ± 1.80	
FT_4_↓	n		1	0	1	2	Fisher *P* = .270
	Mean ± SD		1.78		0.83	1.37 ± 0.76	
FT_4_↑	n		1	1	0	2	Fisher *P* = 1.000
	Mean ± SD		1.78	1.91			
TSH↑	n		1	4	0	5	Fisher *P* = .475
	Mean ± SD		12.89	14.63 ± 8.93		14.28 ± 7.77	
TSH↓	n		0	2	0	2	Fisher *P* = .660
	Mean ± SD			0.219; 0.178		0.219; 0.17	
TPOAB↑ n			1	1	0	2	Fisher *P* = 1.000
TG-ab↑ n			3	1	1	5	Fisher *P* = .387

FT_3_ = free triiodothyronine, FT_4_ = free thyroxine, L = low frequency group, SSNHL = sudden sensorineural hearing loss, T_3_ = triiodothyronine, T_4_ = thyroxine, TG-Ab = thyroglobulin antibody, TH = thyroid hormone, TPOAb = thyroid peroxidase antibody, TSH = thyroid-stimulating hormone.

The most frequent abnormality involved decreased T_3_ and FT_3_ levels, which were present in 27.3% and 18.2% in SSNHL. In subgroup L, 8 patients (25.8%) had decreased T_3_ levels, with a mean value of 1.11 ± 0.13 nmol/L, and 4 patients (12.9%) had decreased FT_3_ levels (1.84 ± 0.18 pg/mL). In subgroup T, 14 patients (33.3%) had decreased levels of T_3_, with a mean value of 1.01 ± 0.16 nmol/L. Additionally, 11 patients (26.2%) in this group had decreased levels of FT_3_ (1.68 ± 0.25 pg/mL), also below reference. In subgroup H, only 2 patients (13.3%) exhibited low T_3_ (1.20 ± 0.85 nmol/L), and 1 patient (6.7%) had low FT_3_ (1.87 pg/mL). A significant difference was observed only in FT_3_ levels between subgroup L and subgroup T (*P* < .05), suggesting a frequency-specific variation in free T_3_ reduction.

Although fluctuations in other thyroid indices (T_4_, FT_4_, TSH, thyroid peroxidase antibody, and TG-ab) were detected across subgroups, Fisher’s exact test revealed no statistically significant differences for these parameters.

### 3.7. Disease classification of TH anomalies in SSNHL

**Figure 1.**
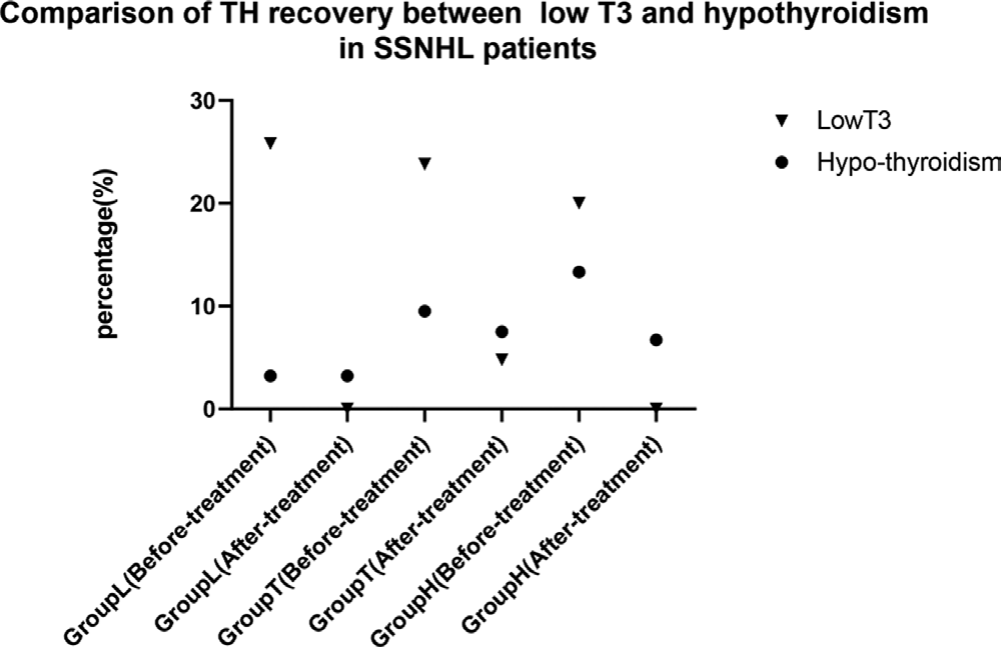
Comparison of TH recovery between low T_3_ and hypothyroidism in patients with SSNHL. SSNHL = sudden sensorineural hearing loss, T_3_ = triiodothyronine, TH = thyroid hormone.

Twenty-one patients in the 3 groups were diagnosed with low T_3_ syndrome, including 8 patients in subgroup L, 10 patients in subgroup T, and 3 patients in subgroup H. The TH level in 19 patients returned to normal after treatment. Seven patients were diagnosed with hypothyroidism, including 1 patient in subgroup L, 4 patients in subgroup T, and 2 patients in subgroup H. The TH level in 2 patients returned to normal after treatment. No differences were observed between the incidence of low T_3_ syndrome and hypothyroidism in the 3 subgroups, and the self-healing trend of the TH level appeared to be more likely in patients with low T_3_ syndrome than in patients with hypothyroidism (*P* < .05; Fig. 1).

### 3.8. Results of multinomial logistic regression

SSNHL in subroup T was selected as the reference category. There were significant differences between subgroup L and subgroup T in terms of variables of sex and age. The value of the regression coefficient of sex (male) was −1.706 and the OR value was 0.182, which meant that females were more likely to experience low frequency HL. The age regression coefficient value was −0.095 and the OR value was 0.909, which meant that young patients were more likely to appear in the low frequency group. However, there was no significant difference in terms of the low T_3_ variable, which meant that low T_3_ was not a factor related to the type of HL (Table 3).

**Table 3:** Results of multinomial logistic regression.

Hearing loss profile		β	*P*	Exp (β; odds ratio)
Low frequency (group L)	Intercept	4.417	.001	
	Sex	−1.706	.011	0.182
	Age	−0.095	.000	0.909
	Low T_3_	1.107	1.674	1.230
High-frequency (group H)	Intercept	−0.250	.859	
	Sex	−0.303	.632	0.738
	Age	−0.029	.190	0.971
	Low T_3_	1.107	.196	3.026

*Notes:* Group T (hearing loss profile of total-frequency) was used as the reference category in the multinomial logistic regression model. Covariates included: sex, age and low T_3_ levels.

OR = odds ratio, T₃ = triiodothyronine.

## 4. Discussion

Endocrine disorders has long been associated with mood and sleep disturbances, as alterations in TH levels can disrupt neuroendocrine homeostasis and central metabolic regulation. Stress-induced activation of the sympathetic nervous system may suppress the hypothalamic–pituitary–thyroid axis, leading to transient reductions in circulating T_3_ and subsequent emotional instability. In this study, we observed significantly elevated rates of anxiety (42.0%), depression (33.0%), and dyssomnia (51.1%) among SSNHL patients compared to controls. These rates are also notably higher than general population estimates reported in China.^[9–12]^ While these findings support a psychosomatic component in SSNHL, the directionality remains unclear.

Thyroid autoimmunity has been proposed as a potential contributor to inner ear dysfunction, including SSNHL and vestibular symptoms. Prior studies have reported elevated rates of thyroid autoantibody abnormalities such as TPO-Ab and TG-Ab in patients with SSNHL, and some data suggest a link between thyroid autoimmunity and more severe high frequency HL. Additionally, autoimmune inner ear disease, although rare, has been linked to systemic autoimmune conditions such as Hashimoto thyroiditis. Proposed mechanisms include T-cell-mediated responses against inner ear antigens and autoantibody deposition within the cochlear or vestibular structures.^[13]^ However, autoimmune inner ear disease is estimated to account for <1% of SSNHL cases, and in our cohort, autoimmune markers were present in only a small subset of patients.^[14]^ Therefore, while thyroid autoimmunity may play a role in certain individuals, it is unlikely to be a predominant etiological factor in the majority of SSNHL cases.

Previous studies on thyroid dysfunction in hearing disorders have often combined biochemical abnormalities (e.g., hypo- and hyperthyroidism) with morphological thyroid changes (such as goiter or thyroiditis), making it difficult to isolate the specific contribution of altered hormone levels to auditory pathology.^[15,16]^ Moreover, existing data have not clearly distinguished whether observed associations are driven by immune-mediated damage, hormonal deficiency, or both. Our study aimed to disentangle these factors by assessing detailed thyroid profiles alongside emotional and sleep parameters.

In our cohort, 33% of SSNHL patients exhibited abnormal TH profiles, with low T_3_ syndrome being more common than overt hypothyroidism. Across all audiometric subgroups, T_3_ reductions were more common than changes in T_4_ or TSH, typically presenting with values slightly below the normal reference range. Most affected patients were clinically euthyroid and demonstrated spontaneous recovery of T_3_ levels within days, suggesting a transient, self-limiting response rather than sustained endocrine dysfunction. These findings align with the biochemical profile of low T_3_ syndrome, likely reflecting peripheral suppression of T_4_ to T_3_ conversion under acute stress rather than primary thyroid disease. Notably, in the low frequency group, most patients with T_3_ reductions lacked diagnostic criteria for hypothyroidism, and a predominance of female patients was observed, though the role of estrogen remains unclear. Autoimmune markers were present in only a minority of cases, suggesting a limited contribution of thyroid autoimmunity in most patients with SSNHL.

By contrast, patients with confirmed hypothyroidism – particularly those with autoimmune thyroiditis – are likely to benefit from hormone replacement. However, traditional levothyroxine therapy often fails to restore physiologic T_3_ levels. Prior studies have shown that levothyroxine-treated individuals tend to have higher FT_4_, lower T_3_, and elevated FT_4_/T_3_ ratios compared to euthyroid subjects.^[17–19]^ Additionally, persistent circulating thyroid autoantibodies may contribute to vestibulocochlear dysfunction via immune-mediated pathways. In such cases, optimizing T_3_ availability and modulating immune activity may be essential for recovery of inner ear function.

Several limitations of this study must be acknowledged. First, although the total sample size was moderate, subgroup analyses were constrained by small numbers, and no a priori power calculation was performed, potentially limiting the ability to detect subtle between-group differences. Second, the cross sectional, retrospective design also precludes causal inference between thyroid dysfunction, emotional disturbance, and SSNHL. Third, the absence of an intervention arm limits the evaluation of whether T_3_ normalization or supplementation influences clinical outcomes. Additionally, while our findings suggest that routine T_3_ therapy may not be necessary due to the self-limiting nature of low T_3_ syndrome, this conclusion should be interpreted with caution. These limitations highlight the need for larger, prospective randomized controlled trials to determine whether selected patients may benefit from targeted TH intervention.

## 5. Conclusions

TH (particularly T_3_) may have a closer association with SSNHL than thyroid autoantibodies, as evidenced by its frequent reduction and rapid normalization during recovery. These findings suggest that TH alterations, especially transient reductions in T_3_, could reflect an adaptive endocrine response to inner ear stress. Monitoring TH levels in patients with SSNHL may aid in early identification of endocrine involvement, and comprehensive management should also address mood, sleep quality, and underlying thyroid dysfunction. Future research should further elucidate the pathophysiological role of TH – particularly T3 – in cochlear function, microcirculation, and auditory recovery.

## Acknowledgments

The authors would like to express sincere gratitude to all of the colleagues for their valuable support and assistance throughout the study.

## Author contributions

**Conceptualization:** Dachun Dai, Yuehua Qiao, Jie Huang.

**Data curation:** Dachun Dai, Yuehua Qiao, Baihe Zhang, Yating Wang, Jie Huang.

**Formal analysis:** Dachun Dai, Yuehua Qiao, Baihe Zhang, Yating Wang, Mengyuan Zhao, Jie Huang.

**Funding acquisition:** Dachun Dai, Yuehua Qiao, Jie Huang.

**Investigation:** Dachun Dai, Yuehua Qiao, Baihe Zhang, Jie Huang.

**Methodology:** Dachun Dai, Yuehua Qiao, Baihe Zhang, Mengyuan Zhao, Jie Huang.

**Project administration:** Dachun Dai, Yuehua Qiao, Baihe Zhang, Yating Wang, Jie Huang.

**Resources:** Dachun Dai, Yuehua Qiao, Jie Huang.

**Software:** Dachun Dai, Yuehua Qiao, Yating Wang, Jie Huang.

**Supervision:** Dachun Dai, Yuehua Qiao, Baihe Zhang, Mengyuan Zhao, Jie Huang.

**Validation:** Dachun Dai, Yuehua Qiao, Yating Wang, Mengyuan Zhao, Jie Huang.

**Visualization:** Dachun Dai, Yuehua Qiao, Jie Huang.

**Writing – original draft:** Dachun Dai, Yuehua Qiao, Jie Huang.

**Writing – review & editing:** Dachun Dai, Yuehua Qiao, Baihe Zhang, Mengyuan Zhao, Jie Huang.

## References

[1] Costa A Arisio R Benedetto C. Thyroid hormones in tissues from human embryos and fetuses. J Endocrinol Invest. 1991;14:559–68. 1940061 10.1007/BF03346869

[2] Constant EL de Volder AG Ivanoiu A. Cerebral blood flow and glucose metabolism in hypothyroidism: a positron emission tomography study. J Clin Endocrinol Metab. 2001;86:3864–70. 11502825 10.1210/jcem.86.8.7749

[3] Bruno R Aversa T Catena M. Even in the era of congenital hypothyroidism screening mild and subclinical sensorineural hearing loss remains a relatively common complication of severe congenital hypothyroidism. Hear Res. 2015;327:43–7. 25987501 10.1016/j.heares.2015.04.018

[4] Santosh UP Rao MS. Incidence of hypothyroidism in Meniere’s disease. J Clin Diagn Res. 2016;10:MC01–3. 10.7860/JCDR/2016/17587.7759 PMC4948427 27437251

[5] The Chinese Magazine Editor Committee of Otolaryngology Head and Neck Surgery, The Chinese Medical Association Otolaryngology Head and Neck Surgery Branch. Diagnosis and treatment guidelines of sudden deafness (2015). Chin J Otolaryngol Head Neck Surg. 2015;50:443–5.

[6] Hobson CE Alexander TH Harris JP. Primary treatment of idiopathic sudden sensorineural hearing loss with intratympanic dexamethasone. Curr Opin Otolaryngol Head Neck Surg. 2016;24:407–12. 27379547 10.1097/MOO.0000000000000288

[7] Rhee TM Hwang D Lee J-S Park J Lee JM. Addition of hyperbaric oxygen therapy vs medical therapy alone for idiopathic sudden sensorineural hearing loss: a systematic review and meta-analysis. JAMA Otolaryngol Head Neck Surg. 2018;144:1153–61. 30267033 10.1001/jamaoto.2018.2133 PMC6583095

[8] Hamilton M. A rating scale for depression. J Neurol Neurosurg Psychiatry. 1960;23:56–62. 14399272 10.1136/jnnp.23.1.56 PMC495331

[9] Zhao K Xu Y Wang MX Zhou HF. Anxiety and depression in patients with sudden sensorineural hearing loss and its influencing factors [in Chinese]. Lin Chuang Er Bi Yan Hou Tou Jing Wai Ke Za Zhi. 2017;31:1735–9. 29798187 10.13201/j.issn.1001-1781.2017.22.008

[10] Zhao Z Yang S Huang H. Status of anxiety and depression in inpatients with sudden deafness and its influencing factors. Pract J Clin Med. 2020:149–52.

[11] Wang YJ Wang MM Hou ZQ Fang ZM Wang HB. Sleep quality analysis in patients with unilateral idiopathic sudden sensorineural hearing loss [in Chinese]. J Clin Otorhinolaryngol Head Neck Surg. 2018;32:209–13. 10.13201/j.issn.1001-1781.2018.03.013 29775024

[12] Yang L Ma Y Wang X Dong N Feng X. Study on relationship between sleep status and prognosis of patients with sudden sensorineural hearing loss. Chin J Clin Healthc. 2016:150–3.

[13] Das S Bakshi SS Seepana R. Demystifying autoimmune inner ear disease. Eur Arch Otorhinolaryngol. 2019;276:3267–74. 31605190 10.1007/s00405-019-05681-5

[14] Khalessi A Borghei P Khalessi MH. Autoimmune inner ear disease- a clinical viewpoint. Iran J Otorhinolaryngol. 2010;22:111–6.

[15] Kim SY Song YS Wee JH Min C Yoo DM Choi HG Association between SSNHL and thyroid diseases. Int J Environ Res Public Health. 2020;17:8419. 33202999 10.3390/ijerph17228419 PMC7697232

[16] Tsai YT Chang IJ Hsu CM. Association between sudden sensorineural hearing loss and preexisting thyroid diseases: a Nationwide Case-Control Study in Taiwan. Int J Environ Res Public Health. 2020;17:834. 32013113 10.3390/ijerph17030834 PMC7037331

[17] Gullo D Latina A Frasca F Le Moli R Pellegriti G Vigneri R. Levothyroxine monotherapy cannot guarantee euthyroidism in all athyreotic patients. PLoS One. 2011;6:e22552. 21829633 10.1371/journal.pone.0022552 PMC3148220

[18] Ito M Miyauchi A Morita S. TSH-suppressive doses of levothyroxine are required to achieve preoperative native serum triiodothyronine levels in patients who have undergone total thyroidectomy. Eur J Endocrinol. 2012;167:373–8. 22711760 10.1530/EJE-11-1029

[19] Jonklaas J Davidson B Bhagat S Soldin SJ. Triiodothyronine levels in athyreotic individuals during levothyroxine therapy. JAMA. 2008;299:769–77. 18285588 10.1001/jama.299.7.769

